# Role of Lipid Peroxidation-Derived **α**, **β**-Unsaturated Aldehydes in Vascular Dysfunction

**DOI:** 10.1155/2013/629028

**Published:** 2013-05-30

**Authors:** Seung Eun Lee, Yong Seek Park

**Affiliations:** Department of Microbiology, School of Medicine, Kyung Hee University, No.1 Hoegi-dong, Dongdaemun-gu, Seoul 130-701, Republic of Korea

## Abstract

Vascular diseases are the most prominent cause of death, and inflammation and vascular dysfunction are key initiators of the pathophysiology of vascular disease. Lipid peroxidation products, such as acrolein and other **α**, **β**-unsaturated aldehydes, have been implicated as mediators of inflammation and vascular dysfunction. **α**, **β**-Unsaturated aldehydes are toxic because of their high reactivity with nucleophiles and their ability to form protein and DNA adducts without prior metabolic activation. This strong reactivity leads to electrophilic stress that disrupts normal cellular function. Furthermore, **α**, **β**-unsaturated aldehydes are reported to cause endothelial dysfunction by induction of oxidative stress, redox-sensitive mechanisms, and inflammatory changes such as induction of cyclooxygenase-2 and cytokines. This review provides an overview of the effects of lipid peroxidation products, **α**, **β**-unsaturated aldehydes, on inflammation and vascular dysfunction.

## 1. Introduction

Vascular disease, a chronic inflammatory disorder associated with vascular injury due to lipid and protein oxidation [1], is the most prevalent cause of mortality and morbidity in almost all parts of the world [2]. Its etiological factors include an interplay between multiple factors such as hyperlipidemia, hypertension, diabetes, obesity, infection, and smoking [3]. Most of these risk factors cause oxidative stress by increasing the level of reactive oxygen species (ROS) [4]. 

Numerous studies have revealed that lipid peroxidation (LPO) products are associated with the development of inflammation-related diseases, such as chronic obstructive pulmonary disease (COPD) and vascular diseases, (including atherosclerosis, Alzheimer's disease and stroke) [5–8]. The accumulation of LPO products in human tissues is a major cause of cellular and tissue dysfunction that may act as physiological mediators in oxidative stress-related diseases [5, 9]. Among LPO products, reactive *α*, *β*-unsaturated aldehydes are thought to contribute to vascular disease and other oxidative stress-related pathologies by covalently modifying proteins and affecting critical protein functions [10]. These products may also promote atherosclerosis by modifying lipoproteins and can cause cardiac cell damage by impairing metabolic enzymes [11]. In this review, we focus on the molecular evidence supporting the role of *α*, *β*-unsaturated aldehydes generated during the lipid peroxidation in inflammation and vascular dysfunction.

## 2. ***α***, ***β***-Unsaturated Aldehydes

In this review, we concentrate on the role of *α*, *β*-unsaturated aldehydes in vascular disease from exogenous (e.g., cigarette smoke) and/or endogenous (e.g., LPO) sources. *α*, *β*-Unsaturated aldehydes can be generated during inflammation because of LPO which is accelerated by diverse oxidative stressors, such as cigarette smoke-generated ROS, reactive nitrogen species (RNS), and free radicals [12]. During LPO, various ROS/RNS oxidize membrane lipids, particularly the polyunsaturated fatty acids, lead to free radical chain reactions and subsequent formation of byproducts, such as *α*, *β*-unsaturated aldehydes. *α*, *β*-Unsaturated aldehydes are highly reactive and can cause atherogenic and carcinogenic effects by injuring blood vessel walls and by forming DNA adducts, respectively. 

**Figure 1:**
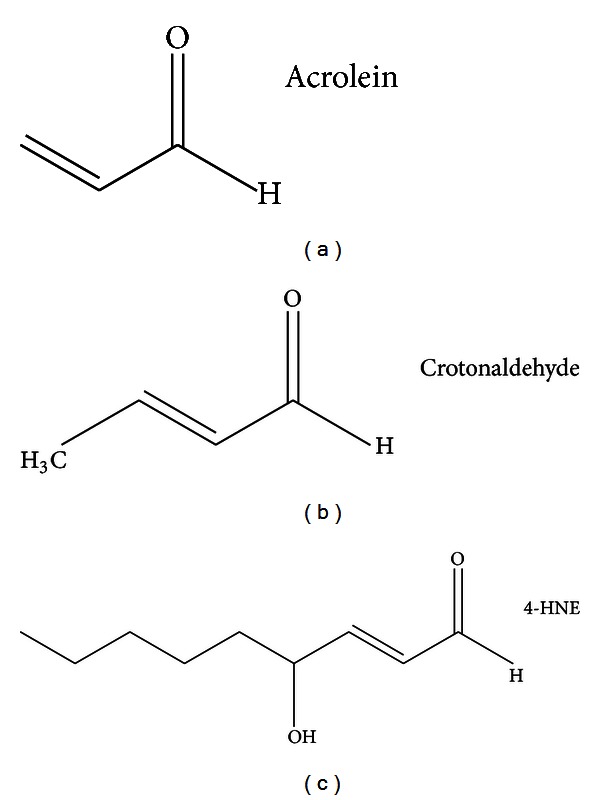
Structures of *α*, *β*-unsaturated aldehydes.

Acrolein (ACR), 4-hydroxy-2-nonenal (4-HNE), and crotonaldehyde (CRA) are the most reactive and toxic *α*, *β*-unsaturated aldehydes (Figure 1). These LPO products can modify nucleophilic side chains on amino acid residues, such as the sulfhydryl groups of cysteine, the imidazole groups of histidine, and the amino acid groups of lysine [13]. Recent studies are reported to the detailed chemistry and the relative electrophilicities of these aldehydes using quantum mechanical parameters [14, 15]. The generation of these strong electrophilic aldehydes and the subsequent adduction of protein nucleophiles may have pathophysiological implications. These aldehydes are associated with elevated tissue levels of their respective protein adducts in disease processes that involve oxidative damage [16, 17]. In addition, the formation of adducts by these reactive aldehydes has been linked to the disruption of cell signaling and mitochondrial dysfunction [14].

### 2.1. Acrolein (ACR)

ACR is present in relatively large amounts (10–140 *μ*g/cigarette) in cigarette smoke and has been implicated in the pathogenesis of vascular disease [18]. ACR is also produced during the incomplete combustion of wood, plastics, gasoline, and diesel fuel; the heating of animal and vegetable fats; and endogenous LPO that is caused by oxidative stress [19]. ACR has a strong electrophilic reactivity towards nucleophiles; therefore, it disrupts the redox control of protein function and causes cytotoxicity via irreversible adduction. In addition, ACR may play a role in the pathogenesis of cardiovascular and neurodegenerative disorders [17]. It is an important oxidative stress biomarker for LPO, and ACR levels increase during aging and in disease, such as atherosclerosis and Alzheimer's disease [20, 21]. Several recent studies have linked ACR exposure to atherosclerosis [22], hypertension [23], dyslipidemia [24], and infarction [25]. 

### 2.2. Crotonaldehyde (CRA)

CRA is abundant in the environment and is also produced endogenously during lipid metabolism [26]. CRA is reported to be present in many foods, such as fish, meat, fruit, and vegetables, and in various liquors [27]. It is formed as a product of LPO and is also produced during the combustion of carbon-containing fuels and other materials [28]; cigarette smoke is another important source of CRA (31–169 *μ*g/kg body weight) [29]. CRA is mutagenic without metabolic activation in numerous cell systems [30] and induces hepatic tumors in rodents [29]. The toxicity of CRA is caused by its strongly reactive electrophilic carbonyl group [31]. Many studies have indicated that CRA directly or enzymatically conjugates with glutathione (GSH), thereby reducing the GSH levels [32]. Previous studies showed that CRA can modulate biological reactions through various downstream signaling pathways and cause cellular oxidative stress [33].

### 2.3. 4-Hydroxy-2-Nonenal (4-HNE)

4-HNE, a strongly reactive *α*, *β*-unsaturated aldehyde, is a diffusible end product of endogenous LPO and is a known marker of oxidative stress. 4-HNE is a potent alkylating agent that reacts with DNA and proteins, thereby generating various types of adducts [31, 34]. These adducts can induce stress signaling pathways and apoptosis [34]. It has been reported that cigarette smoke extract (CSE) causes 4-HNE production either directly or indirectly via LPO in various cell types. In another study by Kode et al. [35], CSE caused a dose-dependent increase in oxidative stress in various cell lines and in 4-HNE levels in small airway epithelial cells (SAECs). CSE-induced cytotoxicity in different cell lines has been attributed to an increase in the endogenous production of 4-HNE. 

Kumagai et al. showed that 4-HNE may be a major inflammatory mediator in the development and progression of atherogenesis [36]. 4-HNE is reported to be producing nerve terminal toxicity by forming adducts that play a critical role in Alzheimer's disease [37]. In addition, studies have revealed that 4-HNE is associated with several other pathological conditions, such as COPD [38], acute respiratory distress syndrome (ARDS) [39], and atherosclerosis [40].

## 3. ***α***, ***β***-Unsaturated Aldehydes in the Pathogenesis of Vascular Diseases

Vascular disease is a complex inflammatory disease that involves several types of inflammatory cells, multiple inflammatory mediators, and oxidative stress. *α*, *β*-Unsaturated aldehydes cause inflammation and damage cells by inducing oxidative stress, redox-sensitive mechanisms, and proinflammatory mediators. The results of many studies have implicated *α*, *β*-unsaturated aldehydes in the pathogenesis of vascular disease (Table 1).

### 3.1. *α*, *β*-Unsaturated Aldehydes and Oxidative Stress

Oxidative stress induced by *α*, *β*-unsaturated aldehydes plays an important role in the pathogenesis of vascular disease through direct injury to the endothelium, as well as through redox-sensitive mechanisms. *α*, *β*-Unsaturated aldehydes increase oxidative stress in endothelial, macrophage, and smooth muscle cells which in turn induces a proinflammatory vascular phenotype by stimulating the transcription of various genes. Cellular oxidative stress and inflammation are implicated in the pathogenesis of many diseases, including stroke, myocardial infarction, and atherosclerosis. Reactive *α*, *β*-unsaturated aldehydes have been shown to induce intracellular peroxide production in endothelial cells [52]. *α*, *β*-Unsaturated aldehydes tend to trigger the formation of ROS or act as oxidants and potentiate oxidative stress in cells [53]. Adams Jr. and Klaidman reported that ACR was oxidized by xanthine oxidase to produce oxygen radicals and that the GSH adduct of ACR also induced oxygen radical formation [54]. ACR depletes endogenous GSH which itself is a critical component of the endogenous antioxidant defense system, thereby increasing the ROS levels [55]. In addition, it has been shown that 4-HNE mediates endothelial nitric oxide synthase (eNOS) uncoupling and superoxide generation by altering tetrahydrobiopterin (BH_4_) homeostasis [56] and that it induces ROS generation by activating nicotinamide adenine dinucleotide phosphate (NADPH) oxidase which is dependent on the activity of 5-lipoxygenase (5-LO) [57]. 

Maintaining the redox balance in the vascular system is of paramount importance since uncompensated oxidative stress contributes to endothelial dysfunction and vascular disease. Oxidative stress is increasingly seen as a major upstream component in the signaling cascade involved in many cellular functions, such as cell proliferation, inflammatory responses, adhesion molecule stimulation, and chemoattractant production. The mechanisms by which endothelial oxidative stress leads to vascular inflammation and the development of atherosclerosis have been reported [58].

### 3.2. *α*, *β*-Unsaturated Aldehydes and Antioxidant Enzymes

Oxidative (electrophilic) stress induces NF-E2-related factor 2 (Nrf2)/antioxidant response element (ARE)-mediated expression of phase II detoxifying and antioxidant enzymes and activates other stress-inducible genes [59]. *α*, *β*-Unsaturated aldehydes are attracted to electrons and can inactivate the nucleophilic active sites of thiolate or selenocysteine enzymes, such as glutathione peroxidase (GPx) through covalent bonding [31]. The inactivation of GPx by *α*, *β*-unsaturated aldehydes is involved in imbalance of the redox state in cell [60]. The thioredoxin (Trx)/thioredoxin reductase (TR) system plays a crucial role in many biological functions, such as redox regulation, apoptosis, and immunomodulation in diverse organisms. Endothelial cells exposed to ACR show rapidly inactivation of TR, resulting in an increase in oxidative cellular damage [52]. In ACR-stimulated human umbilical vein endothelial cells (HUVECs), the induction of heat shock protein 72 (Hsp72) is considered to be a defense system unique to HUVECs [61]. The results of some studies indicate that a highly electrophilic compound, such as ACR, would have the potential to increase Nrf2-mediated gene expression, including that of the cytoprotective antioxidant heme oxygenase-1 (HO-1) in macrophages [62] and endothelial cells [63]. Furthermore, 4-HNE and CRA induces HO-1 expression in endothelial cells [53, 64]. HO-1, a rate-limiting enzyme in heme metabolism, has been recognized as an important factor that protects vascular tissue against atherosclerosis by exerting antioxidative, anti-inflammatory, antiproliferative, anti-apoptotic, and vasodilatory effects on the vasculature. Therefore, increased HO-1 expression in various cells treated with *α*, *β*-unsaturated aldehydes may serve as an adaptive response to oxidative damage.

### 3.3. *α*, *β*-Unsaturated Aldehydes and Inflammation

*α*, *β*-Unsaturated aldehyde-induced toxicity is reported to occur because of depletion of cellular GSH, which subsequently induces ROS production that leads to cell malfunction [55, 65]. ROS was also shown to induce the production of various atherogenic factors, including inflammatory mediators. 

#### 3.3.1. *α*, *β*-Unsaturated Aldehydes and Nuclear Factor Kappa-Light-Chain-Enhancer of Activated B Cells

The nuclear factor kappa-light-chain-enhancer of activated B cells (NF-*κ*B)/Rel family complex is a redox-sensitive transcription factor that plays a role in the expression of various rapid-response genes associated with the inflammatory and immune responses. In addition, NF-*κ*B activation may play a role in the development of chronic inflammatory diseases, such as rheumatoid arthritis, Alzheimer's disease, and atherosclerosis. 

The results of many studies suggest that *α*, *β*-unsaturated aldehydes can regulate inflammation by modulating NF-*κ*B signaling [55]. ACR may affect NF-*κ*B activation, either indirectly by decreasing cellular reduced GSH content or directly by binding to the reactive cysteine on the subunit of I*κ*B kinase (IKK) [55]. The effect of ACR on NF-*κ*B may be cell-type specific and other regulatory mechanisms may also be involved. Li et al. reported that ACR induced I*κ*B expression in rat alveolar macrophage cells, an effect that led to the inhibition of NF-*κ*B activation [66]. However, Haberzettl et al. showed that the ACR-induced increase in cytokine production was accompanied by NF-*κ*B activation [67]. The other *α*, *β*-unsaturated aldehyde, 4-HNE, may also play a role in modulating NF-*κ*B activation through a mechanism similar to that of ACR. It has been suggested that 4-HNE induces 5-LO expression via epidermal growth factor receptor (EGFR)-mediated activation of the NF-*κ*B/extracellular-regulated kinase (ERK) pathways in murine macrophages [68]. 

#### 3.3.2. *α*, *β*-Unsaturated Aldehydes and Proinflammatory Mediators

Cyclooxygenase (COX)-2 is an inducible isoform of COX, which is the key enzyme that regulates the amount of and the duration for which proinflammatory prostaglandins (PG) are produced and also plays a crucial role in inflammation. Under normal conditions, COX-2 expression is tightly regulated, but it is dramatically induced during inflammation by various stimulants. Burleigh et al. suggested that COX-2 expression promotes atherosclerotic inflammation [69]. Since chronic inflammation plays a significant role in atherosclerosis, COX-2 may participate in the development of atherosclerosis. 

The endothelium is a vulnerable target for ACR and related aldehydes. Several studies have reported that exposure to ACR causes endothelial damage [18]. Endothelial cells exposed to ACR exhibit a time-and dose-dependent stimulation of COX-2 expression and enhancement of PG synthesis [21]. The increased PG synthesis in endothelial cells after treatment with ACR reflects an increase in the levels of functional COX-2 protein. In addition, the induction of COX-2 by ACR occurs through activation of the protein kinase C (PKC), p38 mitogen-activated protein kinase (MAPK), and cAMP response element-binding protein (CREB) pathways; it has been suggested that ACR plays an important role in the progression of atherosclerosis via an inflammatory response involving COX-2 expression. 4-HNE is reported to strongly induce COX-2 expression in macrophages [36]. These data suggest that the 4-HNE accumulated in macrophages/foam cells functions as an inflammatory mediator that plays a role in stimulating the inflammatory response and contributes to the progression of atherogenesis.

In addition, Haberzettl et al. showed that ACR treatment increased the production of interleukin-6 (IL-6), tumor necrosis factor-*α* (TNF-*α*), and interleukin-8 (IL-8) in endothelial cells [67]. These findings suggest new proinflammatory and atherogenic aspects of ACR toxicity and the possibility that endogenously produced ACR can contribute toward endothelial injury and inflammation. Because the induction of cytokines, such as TNF-*α*, IL-6, and IL-8, plays a crucial role in atherosclerosis, production of these cytokines may be a significant feature of atherogenesis. Furthermore, ACR treatment induced endoplasmic reticulum (ER) stress and triggered the unfolded protein response [67].

Activated macrophages are reported to generate and secrete matrix metalloproteinase (MMP)-9 which degrades atherosclerotic plaque constituents. A recent study by O'Toole et al. reported that secretion of MMP-9 increases in ACR-stimulated human macrophages [70]. In addition, murine macrophages exposed to ACR exhibited 5-LO overexpression, subsequent proinflammatory leukotriene (LT) accumulation, and enhanced MMP-9 biosynthesis [71]. These data support the possibility that exposure to oxidants or acute inflammatory events can trigger plaque rupture. Akiba et al.showed that 4-HNE accelerates MMP-1 production in human coronary smooth muscle cells (hCSMCs) [72]. MMP-1 (collagenases) cleave native collagen types I and III, which are predominant structural components of atherosclerotic lesions, indicating that increase in the levels of collagenases is a critical event in the progression of atherosclerosis.

## 4. Conclusions 

**Figure 2:**
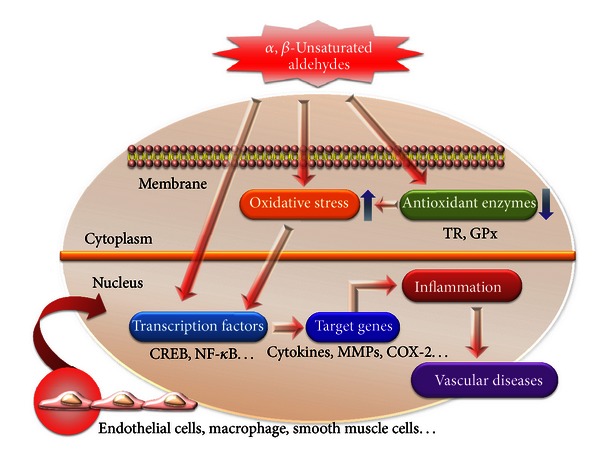
Schematic representation of *α*, *β*-unsaturated aldehydes stimulated leading to inflammation and vascular disease. *α*, *β*-Unsaturated aldehyde reacts directly or indirectly with various genes and transcription factors and induces oxidative stress which may play an important role in inflammation and vascular diseases.

Lipid peroxidation-derived *α*, *β*-unsaturated aldehydes have been shown to play an important pathophysiological role in vascular diseases. *α*, *β*-Unsaturated aldehydes from exogenous and/or endogenous sources, being highly reactive electrophilic molecules, react and modify both proteins and DNA resulting in toxicity. These aldehydes have been implicated in oxidative stress-induced vascular pathologies which act as redox signaling mediators leading to cellular and tissue injury. Furthermore, *α*, *β*-unsaturated aldehydes were reported to induce inactivation of antioxidant enzyme such as GPx and TR, activation of NF-*κ*B signaling pathway, and stimulation of inflammatory response through activation of the proinflammatory signaling pathway (Figure 2). Together, results of these studies provide a better understanding of the involvement of LPO-derived *α*, *β*-unsaturated aldehydes in vascular dysfunction and their possible role in vascular disease. Understanding the mechanism of inflammation-related vascular dysfunction mediated by LPO-derived *α*, *β*-unsaturated aldehydes may help in revealing the pathological factors responsible for vascular diseases and in developing effective therapeutic strategies for these diseases. 

## Figures and Tables

**Table 1:** *α*, *β*-unsaturated aldehydes and vascular diseases.

*α*, *β*-Unsaturated aldehydes	Diseases	References
Acrolein (endogenous/exogenous)	Alzheimer's	Lovell et al., 2001 [41], Bradley et al., 2010 [42]
	Diabetes	Uesugi et al., 2004 [43], Grigsby et al., 2012 [44]
	Atherosclerosis	Uchida et al., 1998 [19], Srivastava et al., 2011 [45]
	COPD	Wang et al., 2009 [46]

Crotonaldehyde (endogenous/exogenous)	Alzheimer's	Kawaguchi-Niida et al., 2006 [28]
	COPD	Volpi et al., 2011 [47]

4-HNE (endogenous/exogenous)	Alzheimer's	Tsirulnikov et al., 2012 [48], Butterfield et al., 2010 [37]
	Ischemia	Eaton et al., 1999 [49]
	Atherosclerosis	Leonarduzzi et al., 2005 [50], Kumagai et al., 2004 [36]
	COPD	Rahman et al., 2002 [38], Halliwell and Poulsen 2006 [51]
